# Alternative methods for the *Plasmodium falciparum* artemisinin ring-stage survival assay with increased simplicity and parasite stage-specificity

**DOI:** 10.1186/s12936-016-1148-2

**Published:** 2016-02-17

**Authors:** Whitney A. Kite, Viviana A. Melendez-Muniz, Roberto R. Moraes Barros, Thomas E. Wellems, Juliana M. Sá

**Affiliations:** Laboratory of Malaria and Vector Research, National Institute of Allergy and Infectious Diseases, National Institutes of Health, 12735 Twinbrook Parkway, Room 3E − 10, Rockville, MD 20852 USA; Universidad Central del Caribe School of Medicine, Bayamon, PR 00960 USA

**Keywords:** *Plasmodium falciparum*, Artemisinin, In vitro anti-malarial assays

## Abstract

**Background:**

Artemisinin-based combination therapy is recommended to treat *Plasmodium falciparum* worldwide, but observations of longer artemisinin (ART) parasite clearance times (PCTs) in Southeast Asia are widely interpreted as a sign of potential ART resistance. In search of an in vitro correlate of in vivo PCT after ART treatment, a ring-stage survival assay (RSA) of 0–3 h parasites was developed and linked to polymorphisms in the Kelch propeller protein (K13). However, RSA remains a laborious process, involving heparin, Percoll gradient, and sorbitol treatments to obtain rings in the 0–3 h window. Here two alternative RSA protocols are presented and compared to the standard Percoll-based method, one highly stage-specific and one streamlined for laboratory application.

**Methods:**

For all protocols, *P. falciparum* cultures were synchronized with 5 % sorbitol treatment twice over two intra-erythrocytic cycles. For a filtration-based RSA, late-stage schizonts were passed through a 1.2 μm filter to isolate merozoites, which were incubated with uninfected erythrocytes for 45 min. The erythrocytes were then washed to remove lysis products and further incubated until 3 h post-filtration. Parasites were pulsed with either 0.1 % dimethyl sulfoxide (DMSO) or 700 nM dihydroartemisinin in 0.1 % DMSO for 6 h, washed twice in drug-free media, and incubated for 66–90 h, when survival was assessed by microscopy. For a sorbitol-only RSA, synchronized young (0–3 h) rings were treated with 5 % sorbitol once more prior to the assay and adjusted to 1 % parasitaemia. The drug pulse, incubation, and survival assessment were as described above.

**Results:**

Ring-stage survival of *P. falciparum* parasites containing either the K13 C580 or C580Y polymorphism (associated with low and high RSA survival, respectively) were assessed by the described filtration and sorbitol-only methods and produced comparable results to the reported Percoll gradient RSA. Advantages of both new methods include: fewer reagents, decreased time investment, and fewer procedural steps, with enhanced stage-specificity conferred by the filtration method.

**Conclusions:**

Assessing *P. falciparum* ART sensitivity in vitro via RSA can be streamlined and accurately evaluated in the laboratory by filtration or sorbitol synchronization methods, thus increasing the accessibility of the assay to research groups.

**Electronic supplementary material:**

The online version of this article (doi:10.1186/s12936-016-1148-2) contains supplementary material, which is available to authorized users.

## Background

In accordance with the World Health Organization (WHO) guidelines, artemisinin-based combination therapy (ACT) is now the front-line treatment for *Plasmodium falciparum* [1]. Increased awareness of prolonged parasite clearance times (PCTs) following artemisinin (ART) treatment in the Greater Mekong subregion has raised concern of potential ART resistance [2–4]. Biological studies of the PCTs and their in vitro surrogates will improve understanding of the mechanisms involved and their impact on parasite fitness [5, 6].

Unlike typical anti-malarial resistance, delayed parasite clearance is not associated with a substantial change in ART 50 % inhibitory concentrations (IC_50_) [3, 7, 8], evaluated by exposing parasites in vitro to serial dilutions of drug [9, 10]. In vitro studies have shown that response to anti-malarials, such as ART, is largely dependent upon intra-erythrocytic parasite stage, as trophozoites and schizonts prove highly susceptible to ART regardless of in vivo phenotype [11, 12]. However, ring-stage parasites aged 0–3 h show differential ability to survive pulses of ART in vitro among genetically distinct parasites [8, 13, 14]. Accordingly, a ring-stage survival assay (RSA) was developed to distinguish parasite survival of an ART drug pulse in vitro. Use of the RSA led to the association of in vitro survival, prolonged PCT, and parasite polymorphisms in Kelch propeller protein (K13) [15, 16]. While ACT is still efficacious, resistance to partner drugs and ART monotherapy place increasing pressure on ART [17], thereby necessitating efforts to investigate the developing relationship of RSAs, K13 polymorphisms, PCTs, and ACT anti-malarial efficacy as thoroughly as possible [18].

RSAs generally rely on a method using heparin, a Percoll gradient, and sorbitol to enrich for 0–3 h ring-stages. Rings are then pulsed with 700 nM dihydroartemisinin (DHA, active metabolite of ART) for 6 h, washed, and grown for 66 h, at which time survival is assessed by microscopy [8]. While effective, this protocol includes multiple steps over many hours, involving several chemical preparations and significant volumes of parasites. Here two alternative RSA preparations are presented: one focused on higher stage-specificity and another on increased time and resource efficiency. The first uses the precision of schizont filtration to isolate merozoites [19], which, after incubating with erythrocytes (red blood cells, RBCs), result in a highly synchronous 3 h ring-stage culture. The second uses sorbitol synchronization only and significantly lessens time investment, thus reducing the effort of the RSA procedure and allowing several assays to be done in parallel with ease. Accurately and efficiently assessing parasites at a specific age supports investigations to understand ART’s mechanism of action and the ability of *Plasmodium* to develop resistance.

## Methods

### Parasites

To evaluate RSA methods *P. falciparum* lines carrying the K13 propeller polymorphism C580Y, which is associated with prolonged PCT and approximately 10 % RSA survival [15, 16], and K13 propeller C580 lines, known to have less than 1 % viable parasites after RSA [8], were chosen. Parasite line 803 contains the K13 C580Y polymorphism and was isolated from a Cambodian patient presenting an extended PCT while treated with three daily oral doses of 4 mg/kg artesunate (AS) [7]. Clone GB4, which does not contain any polymorphisms in the Kelch propeller domain associated with increased PCTs or RSA survival (K13 C580), was isolated from Ghana prior to widespread use of ART in Africa [20]. Progeny from a genetic cross between 803 and GB4 containing either K13 C580 (clones 36F11, 46G9, 24G11, 39E5, and 34F5) or K13 C580Y (clones 61E8 and 76H10) were included in the analysis, along with the parental lines.

### Culture conditions

Parasite cultures were maintained at 5 % haematocrit between 0.5–3.0 % parasitaemia, in complete media (cRPMI): RPMI-1640 (KD Medical, MD, USA) containing 25 mM HEPES and 50 µg/mL hypoxanthine, supplemented with 0.21 % sodium bicarbonate, 20 mg/L gentamicin, and 1 % Albumax II (Life Technologies, CA, USA). Cultures were incubated at 37 °C under a 90 % N_2_, 5 % CO_2_, and 5 % O_2_ gas mixture. Erythrocytes were purchased from the Interstate Blood Bank (Memphis, TN, USA), passed through a Sepacell R-500 filter (Baxter, Deerfield, IL, USA) to remove leukocytes and platelets, washed, re-suspended in RPMI 1640 medium, and stored at 4 °C until use.

### Percoll gradient ring-stage survival assay

To perform the 0–3 h RSA protocol developed by Witkowski, Amaratunga, and colleagues [8] (henceforth designated as the Percoll gradient RSA), 300 µL of packed parasitized RBCs containing 2–3 % mature schizonts (>10 well-segmented nuclei) were collected from a *P. falciparum* culture previously synchronized twice with 5 % sorbitol (Sigma-Aldrich, MO, USA) dissolved in distilled tissue culture-grade water (Gibco, MD, USA), 10 min, 37 °C, over two parasite intra-erythrocytic cycles. The parasites were then incubated in RPMI-1640 containing 15 U/mL heparin (Sigma-Aldrich), but no other supplements, for 15 min at 37 °C. After incubation, parasites were placed onto a 35/65 % Percoll (GE Healthcare Life Sciences, Pittsburgh, PA, USA) gradient, centrifuged at 3000 rpm for 10 min (966×*g*, Eppendorf Centrifuge 5810, rotor A-4-81, no brake). The inter-phase band of late-stage schizonts was collected and incubated in media with fresh RBCs for 3 h, at 37 °C under 90 % N_2_, 5 % CO_2_, and 5 % O_2_ gas mixture (see Additional file 1). This gradient is slightly modified from the original Percoll gradient RSA, as per recommendation of Chanaki Amaratunga to improve separation (personal communication). After incubation, some schizonts had yet to invade; thus, parasites were treated with 5 % sorbitol again to remove remaining schizonts and isolate 0–3 h ring-stage parasites. The parasite mixture was adjusted to 1 % parasitaemia in 20 µl with fresh packed RBCs, and suspended in 1 mL of cRPMI in a 24-well plate containing either 0.1 % dimethyl sulfoxide (DMSO, Sigma-Aldrich, St. Louis, MO, USA) or 700 nM DHA (Sigma-Aldrich, MO, USA) dissolved in 0.1 % DMSO for 6 h. After drug pulse, cells were washed twice with 10 mL cRPMI with 3 min centrifugation at 2500 rpm (671×*g*, 9 acceleration, 5 de-acceleration, Eppendorf Centrifuge 5810, rotor A-4-81). Parasites were further incubated with drug-free cRPMI for 66 h, when thin blood smears were prepared, methanol fixed, and stained with 20 % Giemsa (Sigma-Aldrich, MO, USA) for 15 min for independent survival assessment by at least two experienced microscopists. Evaluation consisted of counting the number of parasitized cells in an estimated 10,000 RBCs and comparing survival to DMSO drug-free incubation.

### Filtration ring-stage survival assay

**Fig. 1:**
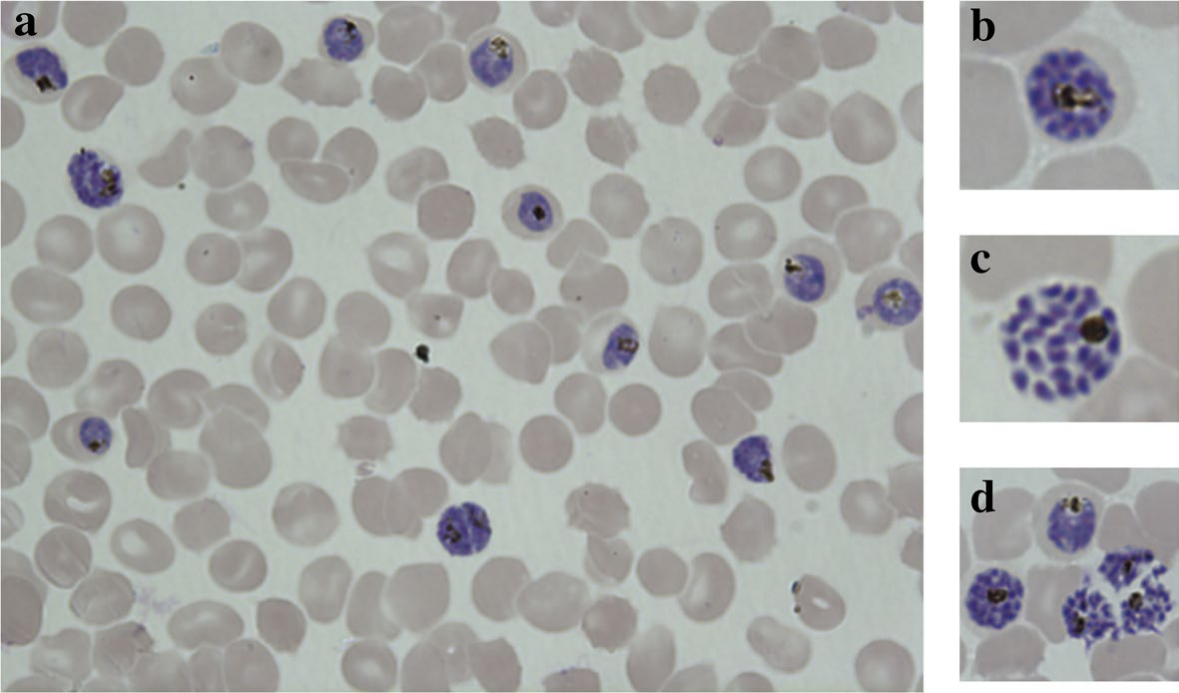
Target morphology of segmented schizonts for filtration. **a** Cultures (1–2 mL packed RBCs) need to be at least 2–3 % mature schizonts for filtration method to work efficiently. **b**–**d** Schizont nuclei are distinct and separated prior to filtration, allowing parasites to burst upon passing through the filter and enter the assay as viable merozoites

**Fig. 2:**
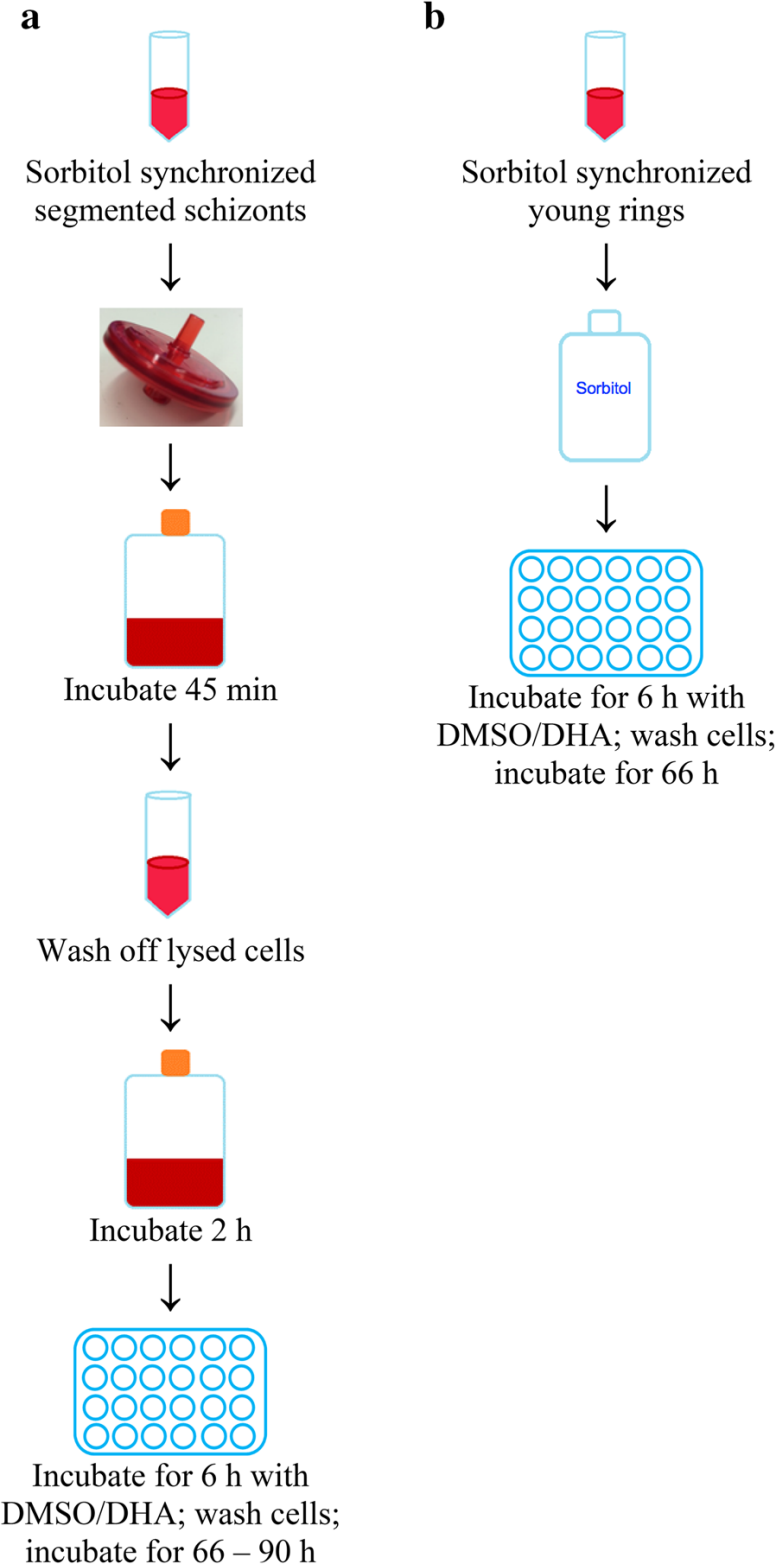
Preparations for drug pulse in filtration and sorbitol-only assays. **a** Steps for filtration RSA are depicted. Late-stage, segmented schizonts are collected, pushed through a 1.2 µm filter, incubated, washed, incubated again, and finally incubated with either 700 nM DHA in 0.1 % DMSO or 0.1 % DMSO only. **b** Sorbitol-only RSA involves a single step. Early post-invasion rings (approximately 3 h old) are collected, synchronized with sorbitol to remove remaining schizonts, and plated at 1 % parasitaemia with either 700 nM DHA in 0.1 % DMSO or 0.1 % DMSO

Parasite cultures were twice synchronized with 5 % sorbitol over two parasite cycles and grown to 40 mL of 5 % haematocrit cultures with at least 2 % parasitaemia. When the majority of parasites were late-stage schizonts, judged as overtly segmented and within minutes of bursting (Fig. 1), cultures were collected, centrifuged at 2500 rpm for 3 min (671×*g*, 9 acceleration, 5 de-acceleration, Eppendorf Centrifuge 5810, rotor A-4-81), combined, and suspended in 10 mL cRPMI. After transferring to a 12 mL syringe, parasites were pushed through a 1.2 μm filter (Pall Corporation, Port Washington, NY, USA) into 200–250 µL fresh RBCs (packed cells) in 10 mL fresh cRPMI [19]. After filtration, the mixture was incubated for 45 min on a plate shaker (speed 3, Boekel Scientific Orbritron V, model 281111), with a 90 % N_2_, 5 % CO_2_, and 5 % O_2_ gas mixture at 37 °C. Cells were then washed with 10 mL cRPMI with 3 min centrifugation at 2500 rpm (671×*g*, 9 acceleration, 5 de-acceleration, Eppendorf Centrifuge 5810, rotor A-4-81) to remove remnants of lysed parasites and RBCs, then further incubated until 3 h post-filtration. Resulting 3 h ring cultures of 0.07–0.15 % initial parasitaemia were adjusted to 2 % haematocrit in 1 mL cRPMI in a 24-well plate containing either 0.1 % DMSO or 700 nM DHA in 0.1 % DMSO and further incubated at 37 °C with gas conditions above (Fig. 2a). After 6 h incubation, cells were washed twice in 10 mL drug-free cRPMI, and then placed in new wells with 1 mL fresh cRPMI for 66 h cultivation. After incubation, thin blood smears of each well were methanol fixed and stained with 20 % Giemsa for 15 min, coded for “blind” parasitaemia assessment, and counted independently by two microscopists, with percent survival adjusted to the DMSO parasitaemia (by counting the number of parasitized cells in estimated 10,000 RBCs). Given the precise age necessary for survival of merozoite filtration, the majority of parasites perish in the filtering process. When too few parasites survive filtration, only low parasitaemias are achieved by 66 h incubation, even in DMSO control wells. In cases of particularly low DMSO parasitaemia (<0.40 %), the cultures were incubated with fresh cRPMI for an additional 24 h and final smears were made at the 96 h time point.

### Sorbitol-only ring-stage survival assay

Parasite cultures were synchronized with 5 % sorbitol twice at exactly 46 h intervals to tighten synchronicity. When parasites were between very late schizonts and very early rings, cultures were synchronized with 5 % sorbitol once more to remove remaining mature forms. Immediately after sorbitol treatment, parasite cultures were adjusted to 1 % parasitaemia in 20 µL RBCs in 1 mL of cRPMI in a 24-well plate, with either 0.1 % DMSO or 700 nM DHA dissolved in 0.1 % DMSO (Fig. 2b). After 6 h incubation, cells were washed and incubated with drug-free cRPMI for 66 h, after which thin blood smears were prepared, methanol fixed, stained with 20 % Giemsa for 15 min and read by at least two microscopists. Percent survival was calculated by counting the number of parasitized cells in estimated 10,000 RBCs and comparing survival to that of the drug-free DMSO incubation.

### Results and discussion

**Fig. 3:**
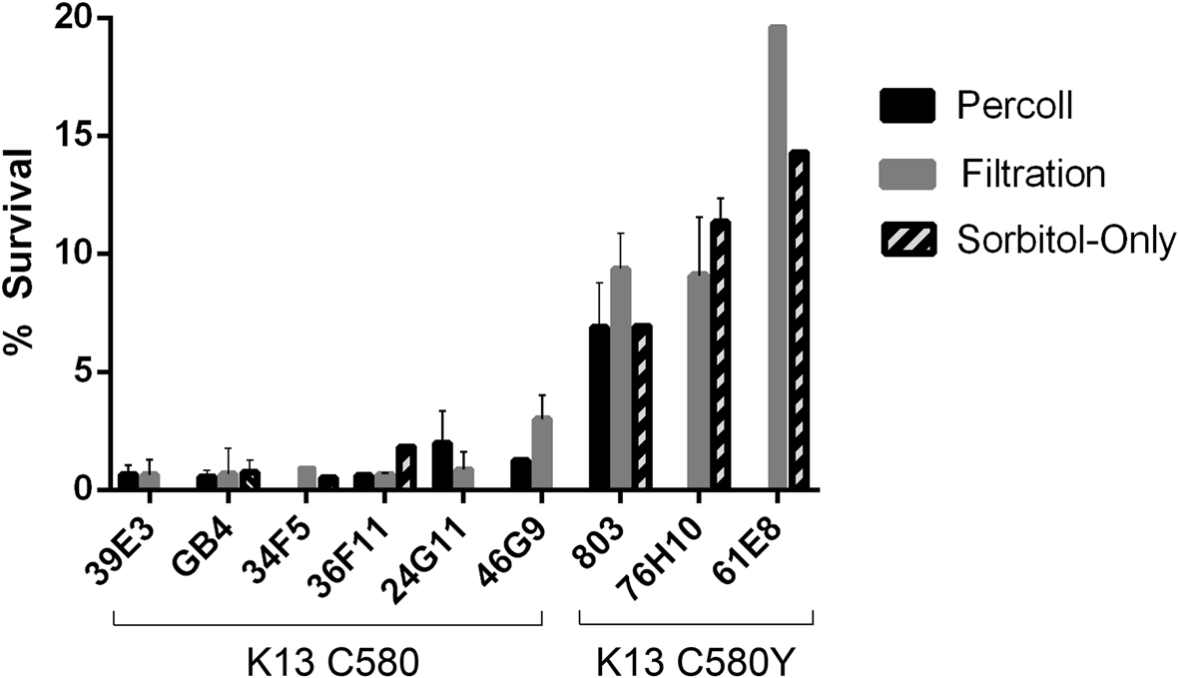
Comparison of results obtained with three RSA methods. The Percoll method is shown in *black*, the filtration method in *gray*, and the sorbitol-only method in pattern. Across the three methods, results are consistent for each line and the distinction between sensitive and surviving is clear. GB4 and progeny 39E3, 34F5, 36F11, and 24G11 all contain K13 C580, while 803 and progeny 76H10 and 61E8 contain K13 C580Y. Survival of all K13 C580 lines is significantly less than those containing the K13 C580Y polymorphism. Values are presented in detail in Additional file 2

As shown in Fig. 3, all three methods produce comparable results, distinguishing survival of parasites with K13 C580 and C580Y. With the Percoll gradient and sorbitol-only methods, <1–2 % of K13 C580 parasites are viable after the DHA pulse, compared to 6–15 % of K13 C580Y parasites. With the filtration method, the ranges of survival increase, with <1–3 % of K13 C580 parasites and as much as 20 % of K13 C580Y parasites persisting after the DHA pulse. Across all three RSA methods, the distinction between high and low survival remains clear.

With all three methods producing consistent outcomes, the methodologies can be directly compared for their respective benefits. Table 1 delineates the distinguishing features of each of the three methods. The two alternative methods presented here eliminate the need for heparin and Percoll. While the required number of parasitized cells is increased fivefold in the filtration protocol, it is reduced tenfold with the sorbitol-only RSA. The filtration RSA may include an additional 24 h incubation in the case of a low initial parasitaemia; however, in response to a significant burden of conducting RSAs, the time required to set up each assay is decreased in both alternative methods.

**Table 1:** Comparison of RSA protocols

Variables	Percoll gradient RSA [8]	Sorbitol-only RSA	Filtration RSA
Chemicals required during RSA^a^	Heparin, percoll, sorbitol	Sorbitol	None
Volume of parasite culture needed	≥300 µL packed RBCs at 2 % parasitemia	40 µL packed RBCs at 1 % parasitemia	≥1.5 mL packed RBCs at 2–3 % parasitemia
Number of steps until completion	17	10	12
Preparation time, before drug pulse	4.5 h	30 min	3 h
Total time of parasite incubation from drug pulse	66 h	66 h	66 or 90 h, if DMSO <0.40 % at 66 h
Stage distribution at time of drug pulse	80 % rings, 20 % trophozoites and schizonts	Mixture of young and older rings	3 h rings only
Presence of pyknotic parasites in read out	Yes	Yes	No

^a^All methods require sorbitol synchronization two parasite cycles before performing RSA

The variation observed between experiments involves several possible confounding factors. In the Percoll and sorbitol-only protocols, variation can be attributed to older rings and even some mature stages surviving 5 % sorbitol synchronization; older rings of sensitive lines have been shown to have decreased sensitivity, and mature stages of both K13 C580 and C580Y lines are highly sensitive to DHA in vitro, thus potentially skewing results [4, 8]. Despite the heightened stage-specificity of the filtration RSA, variation between experiments remains similar to the other methods. This variation could occur because of invasion variability in the 66–90 h post-drug pulse incubation. Measuring survival 24 h after drug pulse would eliminate this variable; however, as the initial parasitaemia of the filtration method can be as low as 0.07 %, measuring survival prior to one or even two rounds of invasion can be difficult.

The large number of pyknotic forms present in the 72 h smears after using the Percoll or sorbitol-only methods may further confound results. Judgment of the viability of the forms by microscopy introduces an additional level of uncertainty and room for error. However, the use of flow cytometry for determination of percent survival may help address this issue [4]. For laboratories without access to flow cytometry, the filtration method eases microscopy assessment as no pyknotic forms are present in the final smears. A possible explanation based on the homogeneity of age distribution suggests highly sensitive and 3 h tightly synchronized rings may simply lyse after drug exposure, whereas slightly older rings present in Percoll or sorbitol-based methods may become pyknotic. These features offer confidence in the results produced by the filtration method, as there is less room for error both in the stage of the exposed parasites and in microscopy assessment.

Others have addressed the need for optimization through highly time-sensitive sorbitol synchronizations [21]. However, while the involved synchronization improves start times for the Percoll assay, it still requires large time investments with over a week of long, irregular hours. In contrast, the proposed alternatives decrease time and resource investment. With the sorbitol-only method, time investment per assay can be decreased to as little as 30 min from collecting parasites to starting the drug pulse. The simpler setup of the assay allows multiple lines to be tested simultaneously with ease, thereby increasing the efficiency of the protocol even further. The 46 h spacing of sorbitol synchronizations is based on the specific in vitro cell cycle of the lines presented here and may not suit the cell cycles of other isolates. Individual laboratories should address the timing of synchronization to align with the cell cycles of their isolates and prevent selection of older rings from isolates with shorter cycles.

Apart from improving RSA stage-specificity, the potential precision of the filtration method opens the door to wider applications of the assay. ART’s mechanism of action may be related to cell stress responses and delayed cell cycles, investigated through time-specific drug pulses and carefully tracking parasite growth after ART exposure [14, 22]. For these assays, accurately staging parasites and maintaining a high level of synchronicity is essential. While estimates of delayed growth are possible through sorbitol synchronized parasites, the stage-specificity of the filtration RSA provides an accurate platform upon which to examine growth-related phenomenon.

## Conclusions

The alternative RSA protocols described here provide simplicity and specificity without compromising the results. The filtration method provides greater stage-specificity, thereby ensuring the age of the parasites is consistent across experiments. The sorbitol-only method provides an alternative to the original method with fewer chemicals, less time, and one-tenth of the parasite culture volume. These methods have not been tested outside the laboratory and thus will need to be validated against the Percoll gradient method in field settings. However, for testing laboratory adapted lines in vitro, the alternative methods provide an opportunity to conduct RSAs with ease and efficiency, thus allowing laboratories with limited time and resources to study ART response in vitro where previously impractical.

## Additional files

10.1186/s12936-016-1148-2. *P. falciparum* late-stage schizont enrichment from blood cultures using 35/65 % Percoll gradient.
10.1186/s12936-016-1148-2. Comparison of RSA results by parasite clone.

## Abbreviations

WHO: World Health Organization
ART: artemisinin and its derivatives
ACT: artemisinin-based combination therapy
PCT: parasite clearance time
IC-_50_: 50 % inhibitory concentration
h: hour(s)
RSA: ring-stage survival assay
K13: Kelch propeller protein
DHA: dihydroartemisinin
cRPMI: complete RPMI 1640 media
RBCs: erythrocytes
min: minutes
DMSO: dimethyl sulfoxide
C: cysteine
Y: tyrosine
