# Supplementary material for: Alternative methods for the Plasmodium falciparum artemisinin ring-stage survival assay with increased simplicity and parasite stage-specificity
Source: Malar J. 2016 Feb 17;15:94. doi: 10.1186/s12936-016-1148-2 (PMC4756417; doi:10.1186/s12936-016-1148-2)
Supplement: Supplementary file 1 — 10.1186/s12936-016-1148-2. P. falciparum late-stage schizont enrichment from blood cultures using 35/65 % Percoll gradient. [file 12936_2016_1148_MOESM1_ESM.docx]

**Additional file 1. *P. falciparum* late-stage schizont enrichment from blood cultures using 35%/65% Percoll gradient.**

| **a** | 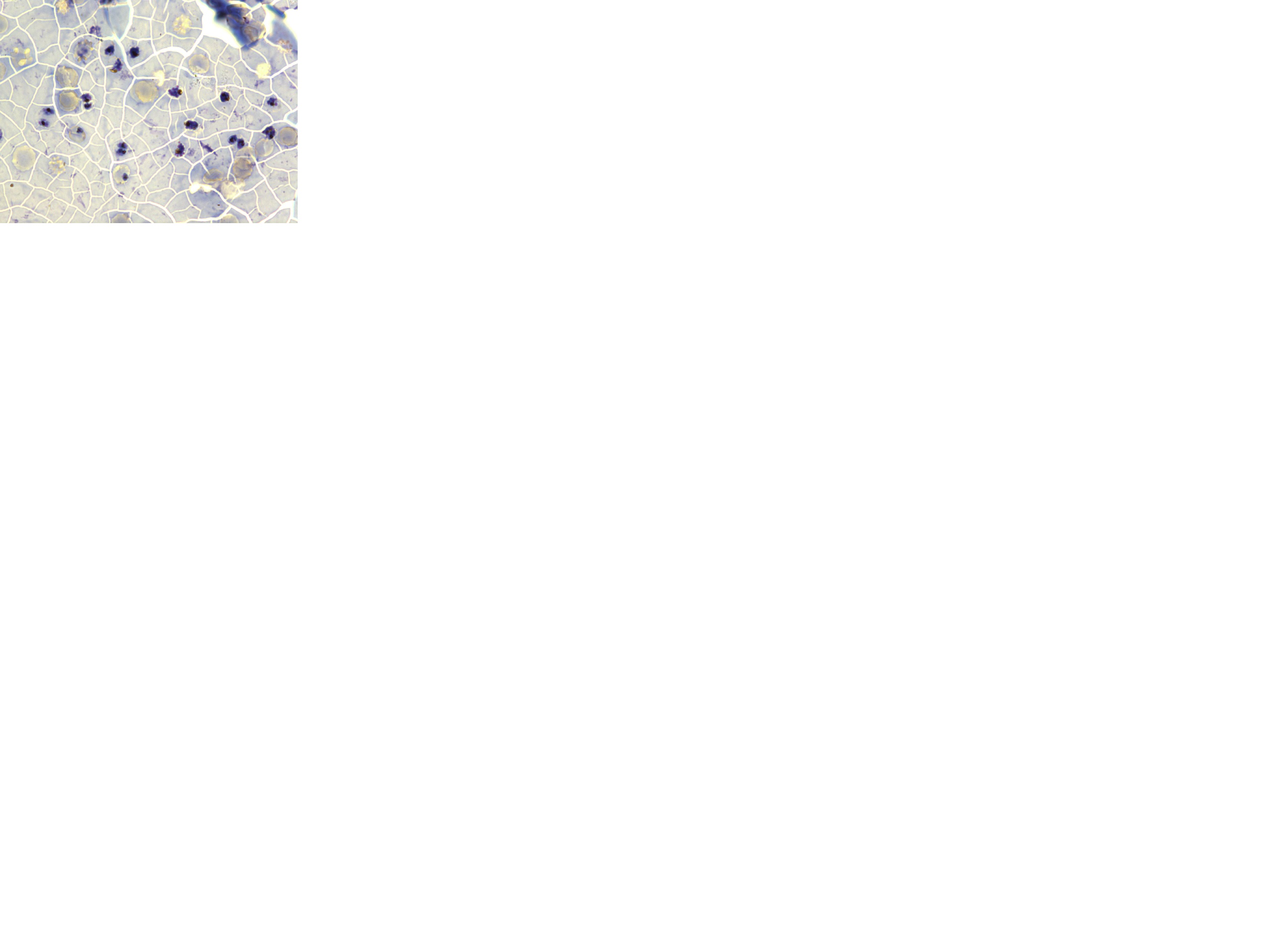 | **b** | 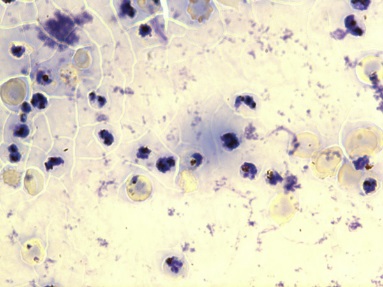 |
| --- | --- | --- | --- |
|  |  | **c** | 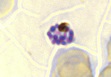 |

Schizonts from the interphase layer of 35%/65% Percoll gradient following centrifugation at 3,000 rpm for 10 min, no brake. **a-b** One microliter of interphase layer fixed with methanol and stained in 20% Giemsa diluted in distilled water for 15 min. Cracking of film is from residual Percoll on slide. 100× oil immersion magnification. **c** Close-up of segmented schizont from the interphase layer.
