# Supplementary material for: Alternative methods for the Plasmodium falciparum artemisinin ring-stage survival assay with increased simplicity and parasite stage-specificity
Source: Malar J. 2016 Feb 17;15:94. doi: 10.1186/s12936-016-1148-2 (PMC4756417; doi:10.1186/s12936-016-1148-2)
Supplement: Supplementary file 2 — 10.1186/s12936-016-1148-2. Comparison of RSA results by parasite clone. [file 12936_2016_1148_MOESM2_ESM.docx]

**Additional file 2. Comparison of RSA results by parasite clone.**

| ***P. falciparum* clone** | **K13 type** | **Parasite survival (%) post-6 h DHA (700 nM) pulse** | | |
| --- | --- | --- | --- | --- |
|  |  | **Percoll Method (n)** | **Filtration Method (n)** | **Sorbitol-Only Method (n)** |
| 39E3 | C580 | 0.66 ± 0.39 (2) | 0.65 ± 0.65 (2) | - |
| GB4 | C580 | 0.55 ± 0.27 (3) | 0.68 ± 1.09 (6) | 0.76 ± 0.50 (5) |
| 34F5 | C580 | - | 0.94 (1) | 0.5 (1) |
| 36F11 | C580 | 0.62 (1) | 0.66 ± 0.04 (2) | 1.82 ± 0.10 (2) |
| 24G11 | C580 | 1.98 ± 1.38 (3) | 0.86 ± 0.75 (3) | - |
| 46G9 | C580 | 1.25 (1) | 3.00 ± 1.03 (2) | - |
| 803 | C580Y | 6.87 ± 1.90 (4) | 8.88 ± 1.52 (9) | 6.92 (1) |
| 76H10 | C580Y | - | 9.05 ± 2.50 (5)^†^ | 11.34 ± 1.01 (3) |
| 61E8 | C580Y | - | 19.63 (1) | 14.29 (1) |

^†^One of the 76H10 filtration experiments was incubated for 96 h. All other experiments were completed at the 72 h time point.

Each line tested by RSA is presented with its K13 580 amino acid (C, cysteine; Y, tyrosine) along with its survival percentage for each method. For each method, the mean percent survival and the standard error (±) of the mean is presented, with the number of experiments conducted in parentheses. A K13 type of C580 is linked to low RSA survival and K13 C580Y is linked to higher RSA survival [16]. The percent survival for each line corresponds to its K13 genotype, with C580Y-containing parasites surviving more than those with C580. Initial parasitaemias were 1% for the Percoll method and Sorbitol-Only method assays and ranged from 0.07-0.15% in the Filtration method assays.
